# Hospitals in India

**Published:** 1893-08-26

**Authors:** 


### HOSPITALS IN INDIA.

### (2) Charitable Dispensaries under the Government of Bengal.

At the end of 1891 there were 272 dispensaries in active operation in Bengal, classified as follows :—

| | | |
|---|---|---|
| Class I. | Government Institutions ... ... ... | 6 |
| Class II. | Dispensaries maintained by Local Funds ... | 139 |
| Class IIIa. | Private Institutions ... ... ... ... | 69 |
| Class IIIb. | Private Institutions aided by Government ... | 58 |
| | Total ... ... ... ... ... ... | 272 |

The available beds numbered 1,682 for male patients, and 619 for female patients. The population of the province is 69,536,861 persons, and it is immediately apparent how inadequate the provision of dispensaries still is. In the Midnapore district, where the population, according to the provisional totals of 1891 was 2,621,750, there were 12 dispensaries, thus giving ·0045 dispensaries per mille of the inhabitants. In the district of the 24 Paraganas, the population was 1,879,920, and the dispensaries numbered 18, or ·0095 per mille of the population. Other districts where the numerical strength of the dispensaries is greatest are Tippera with 15, Mymensingh with 14, Rangpur with 12, and Dacca and Saran with 10 each; but even in these the distribution of dispensaries with regard to population is infinitely too low, and falls altogether too short of the requirements of the province. In other districts, the number of dispensaries is simply insignificant. Thus Backergunge, with a population of 2,147,600, has 4 dispensaries; Monghyr has a similar number and proportion; and Chittagong, Muzaffarpur, and Purnea, have each a proportion of ·0015 dispensaries per mille of the population. Some of these districts, moreover, are feverish and unhealthy.

The total number of indoor and outdoor patients increased from 29,660 and 1,239,850 in 1890, to 35,859 and 1,460,195 respectively in 1891. Taking into account the figures for the Calcutta medical institutions previously referred to, the total number of patients treated in the province in the two years stands as follows :—

| | Indoor. | | Outdoor. | |
|---|---|---|---|---|
| | 1890. | 1891. | 1890. | 1891. |
| Calcutta Medical Institutions ........... | 24,362 | 25,318 | 208,073 | 219,818 |
| Mufassal Dispensaries | 29,660 | 35,859 | 1,239,850 | 1,460,195 |
| Total ...... | 54,022 | 61,177 | 1,447,923 | 1,680,013 |

Compared with the figures of the North-Western Provinces and Oudh for 1890, the above table shows that in Bengal the indoor patients are slightly more numerous, while the number of outdoor patients is considerably less. We append the figures :—

| | N.W.P. and Oudh (1890). | Bengal (1891) |
|---|---|---|
| Indoor Patients......... | 52,566 | 61,177 |
| Outdoor Patients ...... | 2,837,803 | 1,680,013 |

According to the census figures of 1881, the ratio of patients treated to the total population was 21·5 per mille in Bengal in 1891, against 65·5 per mille in the North-Western Provinces and Oudh in 1890. The average daily attendance of indoor patients in 1891 was 1,446·68, and of outdoor patients 10,488·23. Including the figures for the Calcutta medical institutions, the total outdoor attendance for the province was 1,680,013, and the total daily average was 12,160·21, which means that each individual out-patient attended on an average 2·5 times. This appears to be a low average, but it does not differ materially from the average individual attendance in the North-Western Provinces and Oudh in 1890, which was 2·3.

The ratio of deaths per cent. of indoor patients treated during the year was 13·31, against 12·85 in 1890. Cholera and malarial fevers were unusually prevalent during the year, and it was observed that more patients were treated for all diseases during 1891 than during 1890, which may mean either that 1891 was generally a more unhealthy year, or else—which we hope may be the case—that the dispensaries are gradually gaining in popularity, and are being more largely resorted to. In any case the causes of the attendance at the dispensaries, whether it be high or whether it be low, ought to be accurately ascertained. The energy and popularity of the medical officer in charge are known to have increased the attendance at several places, and such men can scarcely be too much encouraged.

The most interesting paragraph in the report we are now considering relates to the attendance of women and children at the dispensaries. Although the number treated as inpatients is still small, it is increasing, and that is the great point. These are the figures for 1891 :—

| | Indoor. | Outdoor. | Total. |
|---|---|---|---|
| Women ...... | 6,223 ... | 222,332 ... | 228,555 |
| Children ... | 2,123 ... | 381,767 ... | 383,890 |

At Burdwan and Bankipur there is fairly suitable provision for the indoor treatment of women, and preparations are being made for the construction of such accommodation in several other places, and the Inspector-General has supplied a standard plan for cottage hospitals to all the Civil surgeons. The Lieutenant-Governor, addressing the committee of the Lady Dufferin Fund in February of last year, said : "The scheme which I intend to propose to the local committee is that we should offer to pay half the salary of a medical lady up to, say, Rs. 30, in every district, and should offer to pay half the cost of erecting a women's ward up to, say, Rs. 1,000, attached to the hospital at the headquarters of every district. When I said women's ward I used the wrong word. It is not a ward that I contemplate, but a row of separate rooms, suited to the secluded habits of respectable women. We have made a great mistake hitherto in several cases in Bengal by creating wards for women on the same pattern as wards for men. No women, but the very poorest, will be content to lie in beds, six, or eight, or ten together in a single ward. We must provide separate quarters for them in which they can retain their privacy, and can be visited and attended by their relatives, and I am glad the Maharaja of Bettiah's hospital is being constructed on this plan. It need not be an expensive plan ; indeed, it should be a less costly one than the construction of wards on the usual system. In this way I hope that in a few years possibly even before I leave this country, I may see a trained lady-doctor and a suitable women's branch hospital in every district of Bengal."

We very gladly give fresh publication to these words, for a perusal of these annual returns of the hospitals and dispensaries in India fills us with a very grave sense of our responsibilities and duties in this direction. Nearly seventy millions of human beings in this Province, and less than three hundred hospitals and dispensaries, many of which have no accommodation whatever for indoor patients ! These are facts, and remarkably ugly facts too.

Including the opening balance, the total income of these dispensaries was Rs. 5,13,726 7a. 7p., and the total expenditure, including investments, was Rs. 4,78,441 4a. 7p. Compared with the returns from the North-Western Provinces and Oudh for 1890 the total cost per patient under all heads taken together, excluding investments, was 5a. 6½p. in Bengal, and 2a. 6½p. in the North-Western Provinces and Oudh. "The fact that Bengal spends twice as much on each patient as its sister Province brings out into singular relief the relative unwillingness in Bengal to make use of the hospitals." This unwillingness is undoubtedly most dispiriting, but ultimately it will assuredly disappear, for steady perseverance and good work always meet with recognition in the long run.

